# Report of a Case of Hemorrhage from the Lungs in a Child 3 Years of Age

**Published:** 1857-04

**Authors:** Hayden Coe

**Affiliations:** Atlanta, Ga.


### ARTICLE IV.

### EXTRACT FROM THE RECORDS OF THE ATLANTA MEDICAL SOCIETY.

E. Hillyer, M. D., Secretary.

*Report of a case of Hemorrhage from the Lungs in a child 3 years of age.* By Hayden Coe, M. D.

On the 9th of February, 1857, about six o'clock in the evening, I was called to a child, M, aged three years.

Her mother informed me that she had had a bad cold for several days, and she thought she was threatened with croup, consequently, she had given her some Hive syrup, which was repeated once after I saw her. Although I did not see any symptoms of croup, there was a little hoarseness and a slight cough, and a little fever, with thin white fur on the tongue. I was called early next morning to see her, and was informed that she had been very sick all night; had vomited several times, and had several operations from her bowels, and that she still was unable to retain any thing on her stomach, not even water. I found her restless, pulse small, quick, fever, thirst, tongue covered with red points, but little or no cough.

I applied a Clove plaster over the stomach, and prescribed alterative powders, Aqua Camphora, Lemonade and ice. This relieved the vomiting in the course of thirty-six hours, but the Diarrhœa continued, and I was unable to control it for six or seven days. She had a good deal of fever, and became very much prostrated, the pulse rapid and feeble; there was a little dry cough during this time, but so slight that it received no notice from me. At one time there seemed to be considerable irritation in the meninges of the brain, so much so the child seemed quite delirious. But a leech was applied to the temple, and small blisters behind her ears, which relieved her head. After the Diarrhœa was checked, the fever soon subsided, and about the twelfth day, the child, to all appearance, was convalescent. The patient, during the whole time, being sustained by water and milk, with flour cooked in it, and a little brandy added to it.

About this time, the submaxillary gland commenced swelling, and it suppurated in a few days and opened and discharged a large quantity of pus into the mouth.

I now ceased to visit my patient, thinking it unnecessary. In four or five days I was requested to prepare an expectorant for her, as her cough had become more troublesome, and that the glands still continued to discharge pus into the mouth. I prepared the expectorant but did not see her.

In a few days after this, I was again requested to visit my patient, as she was thought to be on the decline. To my surprise I found her much emaciated, pale, eyes faded, and was informed that the gland was still discharging freely, and with a troublesome cough. On examining the chest, the whole of the upper portion of the left lung appeared to be in a state of suppuration. In fact, the sounds were similar in every respect to those which occur in the last stage of Phthisis Pulmonalis. This was on the 23d day of February, or about 25 or 26 days from the first attack. I applied a blister over the left lung, and put her on the use of Iodide of Potassium in Sarsaparilla Syrup, and Iron by Hydrogen.

From this treatment, there appeared to be some improvement. But on the 12th of February, about one o'clock at night, she was discovered to be bleeding from the lungs. She died in about 20 minutes after the hemorrhage was discovered.

With the exception of an attack of sore eyes in her infancy, which was manifestly scrofulous, she had very little the appearanee of a child with a scrofulous constitution.

Her parents are healthy and stout in appearance, but her grand mother has had several attacks of hemorrhage from the lungs, although she has lived to be fifty or sixty years of age. Several of her father's relations have also died of Phthisis Pulmonalis.

The hemorrhage must have occurred from either a common abscess or softening of tubercles, which, owing to the chest not being more minutely examined in the earlier part of the disease, cannot perhaps, now be determined with certainty, as a post-mortem could not be had. But I am much inclined to believe it to be the result of softening of tubercles as there were very few symptoms of Pneumonia during the whole course of the disease. The apex of the lungs were most diseased; if it had been an abscess, it would have been most likely to have been situated in the base of the lungs. The suppuration was much too high for an abscess, unless it had been very large. I have but little doubt but that the tubercles existed previously; or at least a predisposition to deposit tubercles, and the great prostration produced by the Diarrhœa and fever, favored the degeneration, as frequently occurs after typhoid fever in colder climates.

If the disease is hereditary, I know no reason why tubercles may not be developed in a child as in the adult, although I have not seen a case where it was so manifest in a young child as in this case.

